# Progress in Disease of Digestive Organs

**Published:** 1902-02-15

**Authors:** 


### Progress in Disease of Digestive Organs.

**Dysentery.**—S. Flexner[1] sums up the results of recent investigations in different parts of the world, and points out that Shiga's bacillus has been found by additional observers in Porto Rico and the Philippines, and kindred bacilli in Germany by Kruse. After careful comparative tests he concludes that the bacilli from various countries are one and the same, and that they are all agglutinated by the blood of convalescent patients, but not by typhoid blood. Washbourn[2] finds that the acute gastro-enteritis common to newcomers in South Africa is often confounded with true dysentery. The former presents an acute, often painless, diarrhœa with liquid stools and rarely a little blood or vomiting. It may occur in sudden epidemics. He divides cases of true dysentery into three classes (*a*) The common type with six days' pyrexia shows constant tenesmus and slimy blood-stained stools, becoming normal about the tenth day, though relapses not infrequently occur even after several months. (*b*) The typhoid type may commence in the ordinary way, but the pyrexia may last three or four weeks, and then liquid offensive stools may appear. There may, indeed, be no intestinal symptoms, and the cases are hard to diagnose from typhoid, though the abdomen is usually retracted. (*c*) The mild type shows no pyrexia, and the usual passage of blood and slime ends in two or three days. As to the complications of dysentery perforation is common, but liver abscess is rare in African cases. We must beware of confounding it with rectal cancer, intussusception, polypi, ulcers due to old fæcal concretions, as well as typhoid. He recommends either the sulphate of magnesium or the ipecacuanha treatment, a diet of plain milk or meat juice, and rectal injections. On convalescence, bismuth and morphia may be needed to stop the simple diarrhœa, and a very careful diet must be taken for some time. He thinks that the so-called asylum dysentery is identical with the African form, but the amœbic or tropical is distinct. W. Rous Kemp[3] prefers a diet of egg albumen to milk, and gives saturated boracic injections night and morning. If the tenesmus is severe a few drops of weak cocaine are applied to the sphincter ani. G. E. Richmond[4] complains that the magnesia treatment is useless in weak patients or those where much ulceration exists, and that with ipecacuanha the patient must be kept in bed and starving to prevent vomiting. Tincture of Monsonia, carbolic acid, or Izal with bismuth are sometimes very useful but uncertain. He reports favourably of 20 grains of sublimed sulphur with five grains of Dover's powder every four hours. The pain and tenesmus disappear very quickly, and, so far as he has seen, relapses do not occur.

**Ulcers.**—Keetley,[5] in reference to malignant ulceration of the large intestines, notices the slowness with which the glands are infected and that the patients are often very stout and do not lose weight for a long while, so that an early diagnosis is most important and yet most difficult. Ulcers may also arise by abrasions from intestinal concretions, gall-stones, foreign bodies, or from the opening of abscesses into the intestines. The cause of chronic ulcers of the colon seems uncertain, but both these and tubercular ulcers often improve greatly after colotomy and local treatment. Among rare causes of ulceration worms must not be overlooked. *Duodenal ulcers* often remain undiscovered. Ladevèze[6] remarks that the hæmorrhage may be either sudden and fatal, or severe and recurrent, or continuous though slight and perhaps unnoticed. Pain is most irregular as to its locality and severity, though most commonly occurring on the right side some three or four hours after a meal. The dyspepsia may be of the hyperacid type or that of chronic catarrh. Jaundice, attacks of dyspnœa and abscess of the liver are very occasionally met with. He recommends rectal feeding for a fortnight when symptoms appear, or surgical treatment may be adopted. Fourteen recoveries out of 16 cases are recorded after operation.

**Appendicitis.**—Matignon, from Pekin,[7] commenting on the absence of the disease in China, seems to think that the use of vegetarian diet may predispose the intestine to resist it. Macewen[8] records two sudden cases where gangrene and perforation were found 30 hours after the first symptom, though there was no doubt that the disease had existed a long while. When operation does not seem immediately needed he gives enemata till the colon is free, and afterwards teaspoonful doses of castor oil till a slight action of the bowel takes place. If the tumour becomes smaller he does not operate till the quiescent stage, and in operations during that stage he has never had a death. He does not advocate the use of saline purgatives though they alleviate the condition, nor does he believe in the recovery of the great majority of patients without surgical aid. F. G. Lloyd[9] thinks that 70 per cent. get well under medical treatment only, but confesses that all forms of the disease may need operation. The *Clinical Review* pleads for the careful use of opium, though opposed by so many surgeons from its obscuring the symptoms.[10] However, against this may be balanced not only the quieting of the patient and of the friends, but the lessened peristalsis, secretion and nervous irritation. Besides, if localised peritonitis has taken place, nothing checks it so well as opium.

**Tuberculous Peritonitis.**—Ulrich Rose[11] contends that too many cases are treated surgically. In the diagnosis of a given case, we must exclude ascites from cirrhosis of the liver, from carcinoma, and from trauma, also a form of ascites seen in children after fevers, and in young girls at puberty. In rare instances, too, ascites occurs in connection with peri-hepatitis, and also in a simple idiopathic form. To distinguish the tuberculous form from these, we must look out for signs of tubercular affections elsewhere, and note the results of the Diazo reaction, of tuberculin, and of injections of the ascitic fluid into guinea pigs, but the latter test often fails from the rarity of bacilli in the fluid. As to the results of operation, he points out that Borchgrevink records a greater percentage of recoveries under medicinal than under surgical treatment, though in selected cases several operators claim over 50 per cent. of recoveries lasting more than two years. Naunyn, out of 71 unselected cases, found that a third recovered without surgical aid, which appears to be quite as good as most surgical results. The treatment under Naunyn was chiefly by fresh air and good diet. Anti-tubercular drugs had some little value, and occasionally calomel or theobronine would remove the fluid. The existence of other tubercular diseases, and above all of phthisis, greatly increased the mortality, but there does not seem, according to Rose, any difference in prognosis between the various forms of the disease, such as the dry or exudative ones. On the whole, if a case improves under treatment, and there are no distinctly surgical complications, we ought to rely on medical treatment alone. Baccarani,[12] too, thinks that the value of surgical aid is over-estimated, and that most of the cases with little fever get well if left alone, and those with high fever are but little helped by it. If there is tubercular infiltration of the bowel causing a stricture with peristalsis and swelling of the right lower quadrant of the abdomen, then an operation is indicated.

**Intestinal Obstruction.**—Bryant [13] divides the causes into (1) those inside the gut, as foreign bodies; (2) changes involving the gut itself, strictures becoming acutely obstructed, intussusception, volvuli; (3) external strangulation by bands or through orifices, internal herniæ, kinking of the gut and tumours compressing it. Under (1) he notices gall-stones and masses of fibre such as those of cinnamon bark and oats. Intussusception is marked by sudden onset, a tumour in the course of the colon, the passage of blood and mucus per rectum in which a tumour may often be felt. The affection is, of course, more common in infants and young children. Volvuli may be formed by a piece of bowel rotated round its long axis or by two pieces twisted round each other. He records one case of volvulus in which a quantity of blood was passed. Strangulation by bands may be due to bands formed by adhesions in peritonitis, or by Meckel's diverticulum, an appendix, a Fallopian tube, the pedicle of a tumour, long appendices epiploicæ, omental cords, or an empty loop of intestine. Between these cases and internal herniæ and acute kinking, a diagnosis is often difficult. There is a sudden onset, early and acute pain, while distension depends on the part of the bowel strangulated. In herniæ of the diaphragm there may be falling in of the abdomen, dyspnœa, displacement of the heart, distension of one side of the thorax with hyper-resonance and loss of the respiratory murmur.

[1] Brit. Med. Jour, September 21. [2] Clin. Jour., August 28. [3] Brit. Med. Jour., August 24. [4] Lancet, June 15. [5] Clin. Jour., August 7. [6] Brit. Med. Jour., May 4. [7] Brit. Med. Jour., April 27. [8] Glasgow Med. Jour. [9] Lancet, September 21. [10] Practitioner, June. [11] Scottish Med. and S. Jour., July. [12] Brit. Med. Jour., May 11. [13] Clin. Jour., June 12.

